# A simple method for lodging resistance evaluation of maize in the field

**DOI:** 10.3389/fpls.2022.1087652

**Published:** 2023-01-04

**Authors:** Jinsheng Yang, Xiangzeng Meng, Shuangyuan Yang, Jinzhong Yang, Zhaoxia Li, Qinglong Yang, Peifeng Zheng, Xiwen Shao, Yongjun Wang, Lichun Wang

**Affiliations:** ^1^ Agronomy College, Jilin Agricultural University, Changchun, Jilin, China; ^2^ Jilin Academy of Agriculture Sciences/Key Laboratory of Crop Eco-Physiology and Farming System in the Northeastern, Institute of Agricultural Resources and Environment, Ministry of Agriculture and Rural Affairs, Changchun, Jilin, China; ^3^ Senior Department, No.1 Middle School Laizhou City Shandong Province, Yantai, Shandong, China; ^4^ Agronomy College, Qingdao Agricultural University, Qingdao, Shandong, China

**Keywords:** maize, lodging resistance, tester, nondestructive evaluation, *in situ*, actual measured value, presumptive value

## Abstract

The increase of planting density is a dominant approach for the higher yield of maize. However, the stalks of some varieties are prone to lodging under high density conditions. Much research has been done on the evaluation of maize lodging resistance. But there are few comprehensive reports on the determination of maize lodging resistance *in situ* without injury under field conditions. This study introduces a non-destructive *in situ* tester to determine the lodging resistance of the different maize varieties in the field. The force value can be obtained by pulling the stalk to different angles with this instrument, which is used to evaluate the lodging resistance of maize varieties. From 2018 to 2020, a total of 1,172 sample plants from 113 maize varieties were tested for the lodging resistance of plants. The statistical results show that the values of force on maize plants at 45° inclination angles (F_45_) are appropriate to characterize maize lodging resistance *in situ* by nondestructive testing in the field. According to the F_45_ value, the maximum lodging resistance F_max_ can be inferred. The formula is: F_max_ =1.1354 F_45_ – 0.3358. The evaluation results of lodging resistance of different varieties of this study are basically consistent with the test results of three-point bending method, moving wind tunnel and other methods. Therefore, the F_45_ value is the optimal index for nondestructive evaluation of maize stalk-lodging resistance under the field-planting conditions.

## 1 Introduction

Maize is the most widespread global food and feed crop. Reasonable densing planting can make full use of natural resources and is the most effective way to achieve high-yield and high-efficiency maize cultivation (Li et al., 2017). Increased maize planting density increases harvest ears and grain yield. However, when the planting density increases beyond a certain extent, lodging is prone to occur (Sun et al., 2021). Maize lodging is extremely harmful to production, not only affecting the stalk character of maize and greatly reducing yield and quality, but also bringing difficulties to field management and harvesting (Deng et al., 2017; Zhang et al., 2018; Sun and Wang, 2020). Therefore, the accurate evaluation of maize stalk lodging resistance has important practical significance for realizing high-yield and high-efficiency cultivation of maize.

Maize lodging generally occurs after the jointing stage, and can be caused by storms, cultivation measures, diseases, insect pests, etc., and especially by strong wind, which causes the maize plants to tilt or fall to the ground after rain (Kamara et al., 2003; Sun and Wang, 2020). The lodging of maize plants can occur in three ways: (1) the bending strength of the stalk is not sufficient to resist the external wind pressure and the stalk is broken (the phenomenon of “stem breaking”); (2) the rigidity of the stalk may be sufficient to resist the external wind force, but the root system has insufficient grip to resist the external wind pressure (the phenomenon of “root collapse”); or (3) the root system has a strong grip and can resist the external wind pressure, and the stalk has poor rigidity and is sufficiently tough that it does not break under the external wind pressure (Robertson et al., 2017), but the inclination exceeds 45° (the phenomenon of “stem lodging”). The lodging degree of maize plants can be divided into mild (tilting 0°–30°), moderate (tilting 30°–60°), and severe (above 85°). Different degrees of lodging have different effects on maize yield. Generally, light lodging reduces yield by 10%–20%, moderate lodging reduces yield by 30%–45%, heavy lodging reduces yield by more than 50%, and even more severe lodging can result in a 100% reduction in yield (Huang and Zhang., 2007; Cheng et al., 2011; Li, 2012). Much research has been done on the evaluation of maize lodging resistance. Laboratory methods of testing stem lodging resistance mainly include the crush test (Zuber and Grogan, 1961; Thompson, 1963), the peel penetration test (Peiffer et al., 2013; Li et al., 2014), and the bending test (Kokubo et al., 1989; Ennos et al., 1993; Li et al., 2003; Gou et al., 2008; Hu et al., 2013; Robertson et al., 2014). In addition, if a correlation between various chemical or morphological factors of plants (e.g., stem diameter, stem lignin content, and stem bark thickness) and the lodging resistance of stems is established, it could be used to predict the lodging resistance of plants. However, these methods usually require a great deal of labor and time, and cannot directly determine the lodging resistance *in situ* of plants in the field. Sibale et al. (1992) measured the puncture resistance of the rind using a modified electronic penetrometer to aid in the selection of plants with higher maize stalk strength. Zhang et al. (2018) evaluated the crushing strength of stems by measuring the force required to break the stems using a hydraulic press. Wen et al. (2019) used a mobile wind turbine to conduct an *in situ* assessment of the stalk lodging resistance of different maize varieties; this research has shown that a new cumulative lodging index (CLI) is more reliable than mechanical properties, failure wind speed (FWS), and wind speed reduction index (RI) when evaluating lodging resistance in terms of reliability and resolution. Cook et al. (2019) invented a maize lodging resistance tester called Darling that breaks the maize stalk by giving it a thrust at a fixed height to obtain the maximum lodging resistance and bending moment, which can evaluate the lodging resistance of maize stalks in the field. Darling is a device capable of inducing a natural destruction and, as such, the device was destructive in assessing maize lodging resistance. Although methods of measuring maize stem strength are becoming convenient and efficient, they are all performed under controlled conditions and can cause some damage to the plant. There are few comprehensive reports on the determination of maize lodging resistance *in situ* without damage under field conditions. When testing the lodging resistance of maize plants from jointing stage to mature stages, it is best to use a non-destructive method and the same population (sample); this can not only objectively evaluate the differences of lodging resistance among different genotypes, but effectively reduce the workload and error caused by different populations (samples). Maize researchers urgently need a technology for the evaluation of maize lodging resistance that can achieve non-destructive *in situ* determination in the field.

Therefore, a simple lodging resistance evaluation method was developed *in situ* for maize plants in the field, which showed simple, fast, efficient and accurate determination of maize lodging resistance. The instrument used the lever principle to pull the maize plants to different angles and measure the real-time pulling force at different angles, which performed the synchronous acquisition of the three parameters of angle, force and displacement (i.e., distance between the main machine and the rotating shaft of the tester). The testing instrument is used to determine the maize lodging resistance using non-destructive measurement *in situ*. The goals of the current study were (1) to establish a simple method for evaluating lodging resistance in the field and (2) to quantify the plant lodging resistance of different maize varieties.

## 2 Lodging resistance tester and testing method

### 2.1 Field experiment design

#### 2.1.1 Experimental design

Field experiments were conducted at the location in Test Site 16, Shandong Denghai Seed Industry Co., Ltd. (E: 119°56.6′, N: 37°20.7′), and Hanting District, Weifang City (E: 119°4.8′, N: 36°53.3′), in Shandong Province, and arranged in 3 years with 113 varieties. The lodging resistance of plants at different planting densities was determined during the flowering period, at the milk ripening stage, and at maturity. The design was shown in **Table 1**. Three replicates were designed for each variety in a location.

**Table 1:** Test varieties in three years.

Years	Locations	Varieties	Test periods	Planting density (plants ha^-1^)	Notes
2018	Test Site 16, Shandong Denghai Seed Industry Co., Ltd., Laizhou, Shandong Province	Denghai661 (DH661)a, Denghai 605(DH605)b, Denghai618(DH618)a, Denghai3622(DH3622)c, Xianyu335(XY335)b, Zhengdan958(ZD958)c	Tasseling stage, milk stage and physiological maturity stage.	4.5, 6.0, 7.5, 9.0, 10.5	The comparisons of lodging resistance at different developmental stages (a: low-ear varieties; b: mid-ear varieties; c: high-ear varieties)
2020	Test Site 16, Shandong Denghai Seed Industry Co., Ltd., Laizhou City, Shandong Province	Denghai605(DH605)b, Denghai618(DH618)a, Xianyu335(XY335)b, Zhengdan958(ZD958)c, Xundan18(XD18)c, Xundan20(XD20)c	Tasseling stage, milk stage and physiological maturity stage.	4.5, 6.0, 7.5, 9.0	The comparisons of lodging resistance at different developmental stages test
2019	Test Site 16, Shandong Denghai Seed Industry Co., Ltd., Laizhou City, Shandong Province	45S01, 45S02, 45S03, 45S04, 45S05, 45S06, 45S07, 45S08, 45S09, 45S10, 45S11, 45S12, 45S13, 45S14, 45S15, 45S16, 45S17, 45S18, 45SCK1, 45SCK2, 45SCK3, Zhengdan958, IY3541, MC588, MC876, NK809, WH1288, ZY303, Chengyu826, Chuangyu188, Dahua1870, Deke501, Denghai125, Guanyu162, Heyu337, Hongsuo899, Huayu688, Jiyu338, Jiyu39, Jidan958, Jinlai318, Jingke9297, Jinnongke445, Jinnongke738, Jiuheyu1, Liyuan296, Luxing617, Mingyu815, Qinliang505, Ruiyou288, Shandan650, Shandan660, Shiyu1502, Tianci1898, Weiyu191, Wugu654, Xianyu1867, Xianyu1871, Xiandai567, Xiandai978, Xundan528, Xianyunuo046, Xianyunuo335, Xiangnong16, Yefeng168, Yongyou988, Yudan188, Yuhong987, Zhaoyu610, Kongfeng191, Zhongbo919, Zhongdan182, Zhongjinyu303.	Physiological maturity stage.	6.75	Regional experimental varieties of maize in Shandong Province
2019	Test Site 16, Shandong Denghai Seed Industry Co., Ltd., Laizhou City, Shandong Province	50S01, 50S02, 50S03, 50S04, 50S05, 50S06, 50S07, 50S08, 50S09, 50S10, 50S12, 50S13, 50S14, 50S15, 50S16, 50S17, 50SCK, 50SCK2, 50CK3, Z50S01, Z50S02, Z50S03, Z50S04, Z50S05, Z50SCK1.	Physiological maturity stage.	7.5	Regional experimental varieties of maize in Shandong Province
2020	Hanting District, Weifang City, Shandong Province	JNK728, DK517, SD650, FK159, FK139, XY047, LP638.	Physiological maturity stage.	7.5	Variety screening test

#### 2.1.2 Meteorological factors

**Figure 1:**
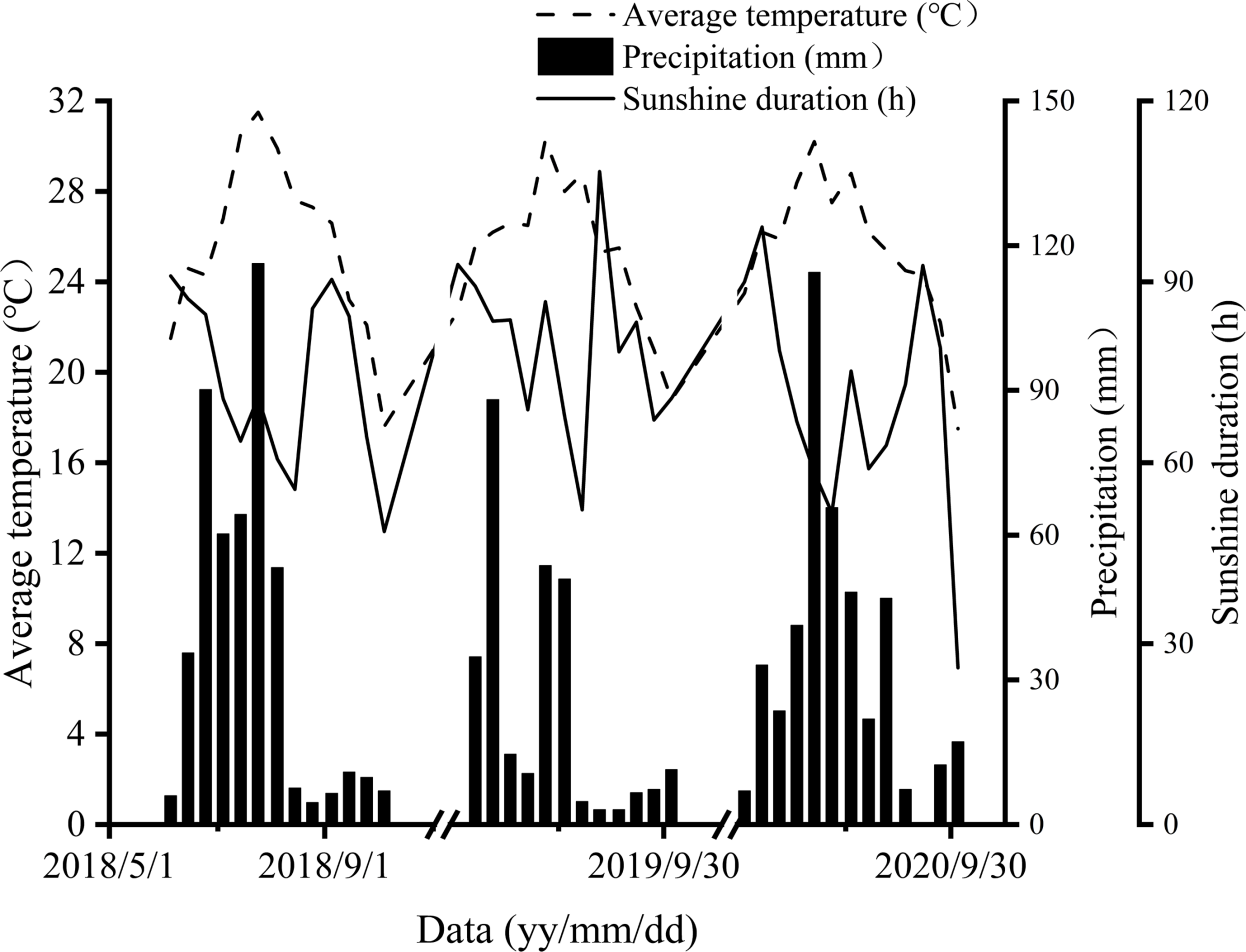
The meteorological factors for 2018–2020.

The meteorological factors for 2018–2020 were given in **Figure 1**.

#### 2.1.3 Agronomy strategy managements

In this study, fertilization was applied according to the yield standard of 11.25 mg ha^–1^, the total fertilization ratio of N:P:K was 3:1:2, and top dressing was applied four times: at the bottom fertilizer stage, at the jointing stage, at the large trumpet stage, and at the male pumping stage. The bottom fertilizer had a N:P:K ratio of 6:6:6, and the remaining phosphate fertilizer and potassium fertilizer were applied once at the jointing stage. Nitrogen fertilizer was applied such that 40% of the total N required was provided at the jointing stage and 50% of the total N required was provided at the large trumpet stage; the remaining nitrogen fertilizer was applied once at the male pumping stage.

### 2.2 Test method

#### 2.2.1 Placement of lodging resistance tester

**Figure 2:**
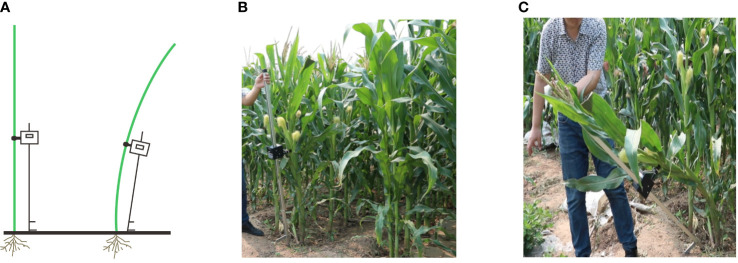
Test process of lodging resistance tester. **(A)** Schematic diagram of instrument placement during a test process. **(B)** Place the instrument parallel to the plant at the beginning of the test. **(C)** Pull the instrument vertical rod by hand for testing.

A sample of maize plants in the field were selected for testing. Plants with similar plant height, ear height and stalk thickness were selected within the population to eliminate the marginal effect on the experiment. The lodging resistance tester was placed in parallel wit the target maize plant and at a fixed distance away from the tester. The pedal was pressed to insert the fork head into the soil for fixation and the fixing nut of the main machine loosened to slide it along the vertical rod to the ear height. The fixing nut again was then tightened again and the stalk of the maize clamped to test (**Figures 2A, B**).

#### 2.2.2 Determination of lodging resistance

##### 2.2.2.1 Maximum lodging resistance determination

The test key was pressed to start the test procedure, the upper part of the tester was manually pulled evenly, and the vertical rod slowly turned, until the plant snapped or pulled to 90°. During the test, the main machine automatically recorded the angle, tension, and displacement. The maximum tension reflected the maximum lodging resistance of the plant.

##### 2.2.2.2 Measurement of lodging resistance non-destructive

The test button was pressed to start the test, the upper part of the tester was pulled by hand, trn the vertical rod was turned at a constant speed to pull the plant until it was tilted at a 45° angle, then the main machine was loosened, and the plant returned to its normal growth state. During the test, the main machine automatically recorded the angle, tension, and displacement (**Figure 2C**) for the determination of lodging resistance.

##### 2.2.2.3 Calculation of expected values

*F*_max_ and *F_45_* were fitted, *y *= *ax *+ *b* (*a* and *b* are constants, *x* was the bending force of the stalk when the stalk was bent 45° (*F*_45_), and the expected value (*y*) is calculated by the fitted equation.

### 2.3 Field non-destructive *in situ* maize lodging resistance tester

#### 2.3.1 Design principle

**Figure 3:**
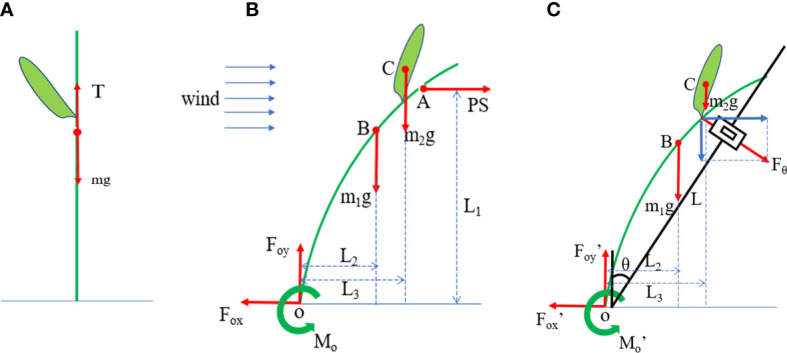
Analysis of maize plant force. **(A)** Stress analysis of maize plants under windless conditions. *T*, ground support force; *mg*, gravity of maize plant. **(B)** Stress analysis of maize plants under the influence of external wind. **(A)**, the concentrated stress point on the windward side of a maize plant; B, the center of gravity of stalk part of maize plant; **(C)**, the center of gravity of ear part of maize plant. *PS*, wind force exerted on maize plant; *m*_1_ *g*, gravity of stalk part of maize plant; *m*_2_g, gravity of ear part of maize plant; *F*_ox_, horizontal force on maize root; *F*_oy_, vertical force on maize root; *L*_1_, arm of force of *PS*; *L*_2_, arm of force of *m*_1_ *g*; *L*, arm of force of *m*_2_ *g*; *O*, stem base; *M_o_*, resistance moment of maize plant. **(C)** Pulling force given to the maize plant by the apparatus during the resistance to overturning test. θ, inclination angle of vertical shaft of instrument, *F*_θ_, tension at inclination angle θ; L, the arm of *F*_θ_, *F*_ox′_, horizontal force on maize root, *F*_oy′,_ vertical force on maize root; *M*_o′,_ resistance moment of maize plant.

In the absence of external wind, the maize plant was supported only by gravity *mg* and ground support *T*, and the two forces reach balance (**Figure 3A**).

Assuming that the wind was horizontal, the wind pressure was *P*, and the windward area of the maize was *S*. If point A was the concentrated stress point on the windward side of the maize, the wind force received by the maize plants at point A was *PS*. When subjected to wind, the plant’s swaying motion can be resolved as a series of motions on the vertical plane with the root system as the origin. Under the combined action of wind *PS* and gravity, *mg*, the plant would be inclined and bent, and the resistance moment *Mo* was produced by the anchoring of the root system. If maize plants are divided into two independent parts, stalk and ear, the mass of the stalk was *m*_1_ and the mass of ear was *m*_2._ Maize plants reached a force equilibrium state under the action of wind, gravity, and self-resistance (**Figure 3B**). At this time, the torque produced by wind and gravity was equal to the bending torque produced by maize plants. The equilibrium equation of its force (Liu, 2017) was as follows:


$$\{\begin{array}{c} PS–F_{\text{ox}}=0\;\;\;\;\;\;\;\;\;\;\;\;\;\;\;\;\;\;\;\;\;\;\;\;\;\;(1)\\ m_{1}g+m_{2}g\;–F_{\text{oy}}=0\;\;\;\;\;\;\;\;\;\;\;\;\;\;\;\;\;(2)\;\\ M_{\text{O}}=PS\cdot L_{1}+m_{1}g\cdot L_{2}+m_{2}g\cdot L_{3}\;\;\;\;(3) \end{array}$$

In the absence of wind, we use the instrument to pull the maize plant to tilt and bent it (**Figure 3C**). The balance equation of its force (Liu, 2017) is as follows:


{Fθ·cosθ–Fox=0                   (4)Fθ·sinθ+m1g+m2g –Foy=0     (5)MO'=Fθ·L+m1g·L2+m2g·L3    (6)

The moment *Mo* of the maize plant resisting the resultant force of wind and gravity was equal to the moment *Mo*′ of the maize plant resisting the pulling force, when we used the instrument to pull the maize plant to tilt to the same degree as when exposed to the wind. Therefore, in the horizontal direction, the moment *PS*∙*L*_1_ of wind was equal to the moment *F*_θ_∙*L* of instrument tension in the horizontal direction of the stem and, in the vertical direction, the moment of gravity “*m*_1_ *g*∙*L*_2 _+ *m*_2_g∙*L*_3_” was constant.

For the same variety, the center of gravity of the stalk part was the same, and the weight moment *m*_1_ *g*∙*L*_2_ of the stem was constant. However, the difference in *L*_3_ was caused by the difference in the height of the ear position. The higher the ear position, the greater the moment *m*_2_ *g*∙*L*_3_ of the ear weight. Because *F*_θ_∙*L* was equivalent to *PS*∙*L*_1_, the windward area *S* is roughly the same, and *L*_3_ was positively correlated with *L*, so *P* was positively correlated with *F*_θ_.

The maximum bending moment was fixed as the same variety. When the critical bending moment *M*_max_ was reached, according to formula (3), the height of ear leads to an increase in *m*_2_ *g*∙*L*_3,_ the corresponding wind power *PS* decreases, and the wind resistance of the maize decreases.

Different varieties had different ear height and their tensile force varies. This change was reflected in the fact that when *Mo*′ was the same, the larger *L*_3_ was, the smaller *F*_θ_ was at the same angle. Because the height of the maize ear was the main influencing factor, the *F*_θ_ produced by the instrument pulling at the ear position could be used to evaluate the wind resistance of maize.

When the wind was low, the maize plants swing back and forth with the root system as the origin. With the increase in wind force, the resultant moment of wind force *PS* and gravity will also increase. At this time, if the anchoring force of root system was weak, root fall would occur. If the anchoring force of the root system was strong but the quality of the stem was poor, plastic deformation would occur, which will cause the stem to break. *Mo*′ at the inclination angle of the plant was the maximum lodging resistance moment of the plant when the root falls or the stem breaks, and *F*_θ_ was the critical lodging resistance force.

The stalk material of the same maize plant was the same, the center of gravity and ear position are the same in the same growth period, and the arm of force *L* is the same. As long as *F*_θ_ was measured, the lodging resistance of the maize stalk can be evaluated. If *F*_θ_ was measured before lodging or folding, the evaluation can be realized without damaging maize plants (**Figure 3C**).

#### 2.3.2 Non-destructive maize lodging resistance tester

**Figure 4:**
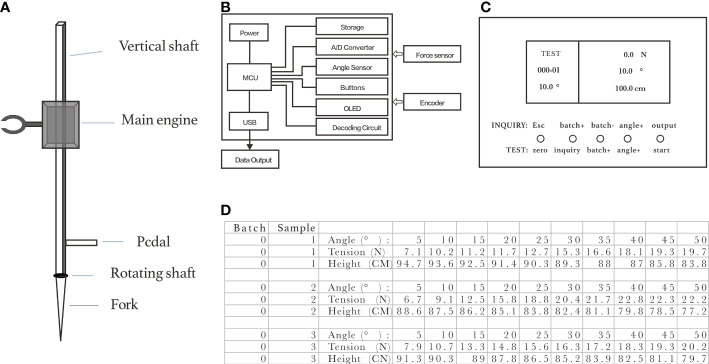
The physical appearance, major components and export data format of lodging resistance tester *in situ* for maize. **(A)** Tester structure: it consists of the main engine, vertical shaft, rotating shaft, pedal and fork head. The vertical shaft is connected to the fork head through the rotating shaft, and the pedal is located at the lower part of the vertical shaft. The test host can slide up and down along the vertical bar. **(B)** Electronic system: the main engine integrates the angle, tension, and height sensors, which is controlled by microcontroller. The angle sensor uses a single-axial gyroscope, output to MCU through I2C port, with accuracy of 0.1°; the tensile sensor adopts an S-type tension integrated weighing sensor, output voltage signal into digital signal to MCU through AD conversion, with accuracy of 0.1 N; the height sensor adopts a displacement encoder, transmitted to MCU, measuring reference to the bottom end of vertical rod rotating shaft, with accuracy of 0.1 cm. Other types of sensors can also be connected to this device. The power supply device comes from the charging lithium battery, which can power the device for more than six hours. **(C)** User interface: consists of a 3.5-inch LCD screen and five selection buttons at the bottom of the device. These five buttons integrate the two functions of preparation mode and query mode. The user interface is written in the C programming language. **(D)** Export data format: each set of data has three group values: angle, force, and displacement (distance between vertical line and the rod of the tester). After the test ends, import the data into the U disk in a XLS file format.

Based on the above idea, Shuangyuan Yang invented the field non-destructive *in situ* lodging resistance tester (NDT) for maize plants, which was commercially produced by Laizhou Kaitian Instrument Co., Ltd. The instrument model was KTDF-1 (**Figure 4**). At present, three patents had been granted, namely, a Chinese invention patent (patent no. ZL201510176119.9), a Chinese utility model patent (patent no. ZL201720355104.3), and a German utility model patent (patent no. 202017106298).

The field non-destructive *in situ* lodging resistance tester for maize (**Figure 4A**) could be used to determine the force and dynamic displacement at different angles, until the maximum bending force that leads to the breaking of maize stalk is found. The tester could be automatically adjusted for displacement measurement. The displacement is produced by the force vector of the plant at different angles. By using the tester, the dynamic determination of pull forces and the changes of angles could be achieved. The main engine of the instrument is controlled by a microcontroller (**Figure 4B**) and operated through the user interface (**Figure 4C**). The force and stalk position obtained at different angles during the test could replace *F*_θ_ (the wind force *PS*). According to Equations (4) and (5), the lodging resistance of maize plants under the force of wind could be evaluated. Data from the test can be imported into a computer for analysis (**Figure 4D**).

### 2.4 Statistical analysis and processing

IBM SPSS Statistics 26 (IBM Corporation, Armonk, NY, USA) was used for data statistics and analysis. Maize varieties were clustered according to the squared Euclidean distance method. Origin 2021 (Origin Lab, Northampton, MA, USA) was used for data processing and plotting. Comparisons among groups were tested by one-way analysis of variance and the least significant difference test, and differences between the means were considered significant at *p*<0.05.

## 3 Results

### 3.1 Evaluation for the criteria of maize lodging resistance by F_max_ values

**Figure 5:**
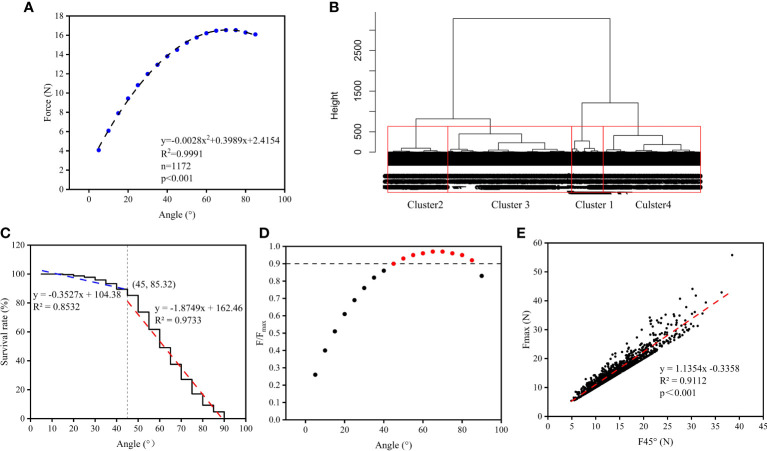
The data analysis of lodging resistance from maize plants at different locations in three years. **(A)** Stress values of maize plants at different angles. **(B)** Clustered lodging level of maize plants according to the tested *F*_max_. **(C)** The survival rate of maize plants under different tilted angles. **(D)** The ratio of *F* to *F*_max_ under different tilted angles. **(E)** Correlation between *F*_45_ and *F*_max_.

From 2018 to 2020, 1,172 maize plants were tested in Laizhou and Weifang Region, Shandong Province, at the flowering, milk ripening, and maturity period. As the plant tilted angle increases, the pull trajectory of the plant determined by a tester complies with the equation y =–0.0028*x* ^2^ + 0.3989*x*+2.4187 (*R* ^2 =^ 0.9991, *n *= 1172), where *x* represents the tilted angle, and *y* represents the pull value at the angle (**Figure 5A**).

The *F*_max_ values of the above maize plants were clustered by Euclidean distance and divided into four categories (**Figure 5B**) with a mean of 9.25 N, 14.53 N, 20.61 N, and 31.33 N, which consisted of 32.25%, 37.88%, 22.70%, and 7.17% in the total number of varieties. Accordingly, the lodging resistance levels of maize was divided into four levels: poor lodging resistance (cluster 3, *F*_max ≤_11.8 N), low lodging resistance (cluster 2, *F*_max_ > 11.8 N but ≤17.5 N), medium lodging resistance (cluster 1, *F*_max_ *>*17.5 N but ≤25.8 N), and high lodging resistance (cluster 4, *F*_max_ *>*25.8 N) (**Table 2**).

**Table 2:** The *F_max_* clustering by the Euclidean distance method.

	*F*_mean_	*F*_min_	*F*_max_	Samples in each cluster
cluster 1	31.33	26	55.8	84
cluster 2	20.61	17.6	25.8	266
cluster 3	14.53	11.9	17.5	444
cluster 4	9.25	3.4	11.8	378

### 3.2 A correlation between *F*_45_ and *F*_max_

As shown in **Figure 5E**, *F*_45_ and F_max_ are strongly correlated, and F_max_ predicted by F_45_ can be used to assess the lodging resistance of maize plants according to the correlation. Simulation results agree well with measurements.

As the tilt angle of the maize plant increased, the breaking ratio of the plants gradually increased. When the tilt angle was below 45°, the breaking ratio was relatively low. When tilt angle was between 5° and 40° the cumulative proportion of breaking plants was 10.41%; at 45° the proportion was 4.27%, and at 50° the proportion was 11.60%, indicating that a plant tilt angle of 45° was the critical point for a significant decrease in the survival rate of plants. The proportion of plants breaking increases slowly as tilt angle increases up to<45°, and the survival rate of plants has a curve slope of –0.3527; when the tilt angle goes beyond 45°, the survival rate of plants decreases quickly, accompanied by a curve slope of –1.8749 (**Figure 5C**).

In the process of the test, as the tilted angle θ of the plant increases, the pull force of the *F* value (expressed as *F*_θ_) increases until the plant breaks or does not break at a 90° angle. Subsequently,the maximum *F* value (expressed as *F*_max_) can be determined. As the results, the larger the tilt angle, the closer to *F*_max_ is *F*_θ_. A map was made by using *F*_θ_/*F*_max_ values and tilted angles (**Figure 5D**). It wis interesting that, at tilt angles in the 15–55° range, th *F*_θ_/*F*_max_ increases proportionally, and *F*_θ_/*F*_max_ is about 0.9 at a 45° tilt angle (**Figure 5D**), which could characterize the lodge resistance of maize plants.

Out of 1,172 tested maize plants, 1,000 tilted by 45° without the stalk breaking and were selected to determine *F*_45_ values and *F*_max_, and comparing *F*_45_ with its corresponding *F*_max_ developed the equation *y* = 1.1354*x* – 0.3358 (*R*² = 0.9112), indicating that a strong correlation exists between *F*_45_ and *F*_max_ (**Figure 5E**). By measuring *F*_45_ and applying the formula, expected values for maximum stalk resistance can be obtained. The maize plants under a 45° tilt angle maintained a 85.32% survival rate in a large complex population; therefore, *F*_45_ can be used to evaluate the lodging resistance of maize plants with a simple method.

### 3.3 Evaluation of lodging resistance for the different varieties of maize

The F_max_ was predicted by F_45_ and the lodging resistance of plants were evaluated under non-damaged conditions. Comparing the measured and expected values in pairs, the results are shown in **Table 3**. In the low-resistance group the accuracy reached 97.28%; in the high-resistance group the accuracy was 88.57%, which was the lowest accuracy (**Table 3**). It was concluded that the two tests produced similar results for the lodging resistance of plants.

**Table 3:** Comparing of the lodging resistance levels of plants by measured and inferred values.

Levels of lodging resistance	Lodge-prone	Low resistance	Moderate resistance	High resistance	Total
Individual distribution from expected values	258	415	249	78	1000
Individual distribution from measured values	248	404	278	70	1000
Accuracy (%)	96.0	97.28	89.57	88.57	

**Figure 6:**
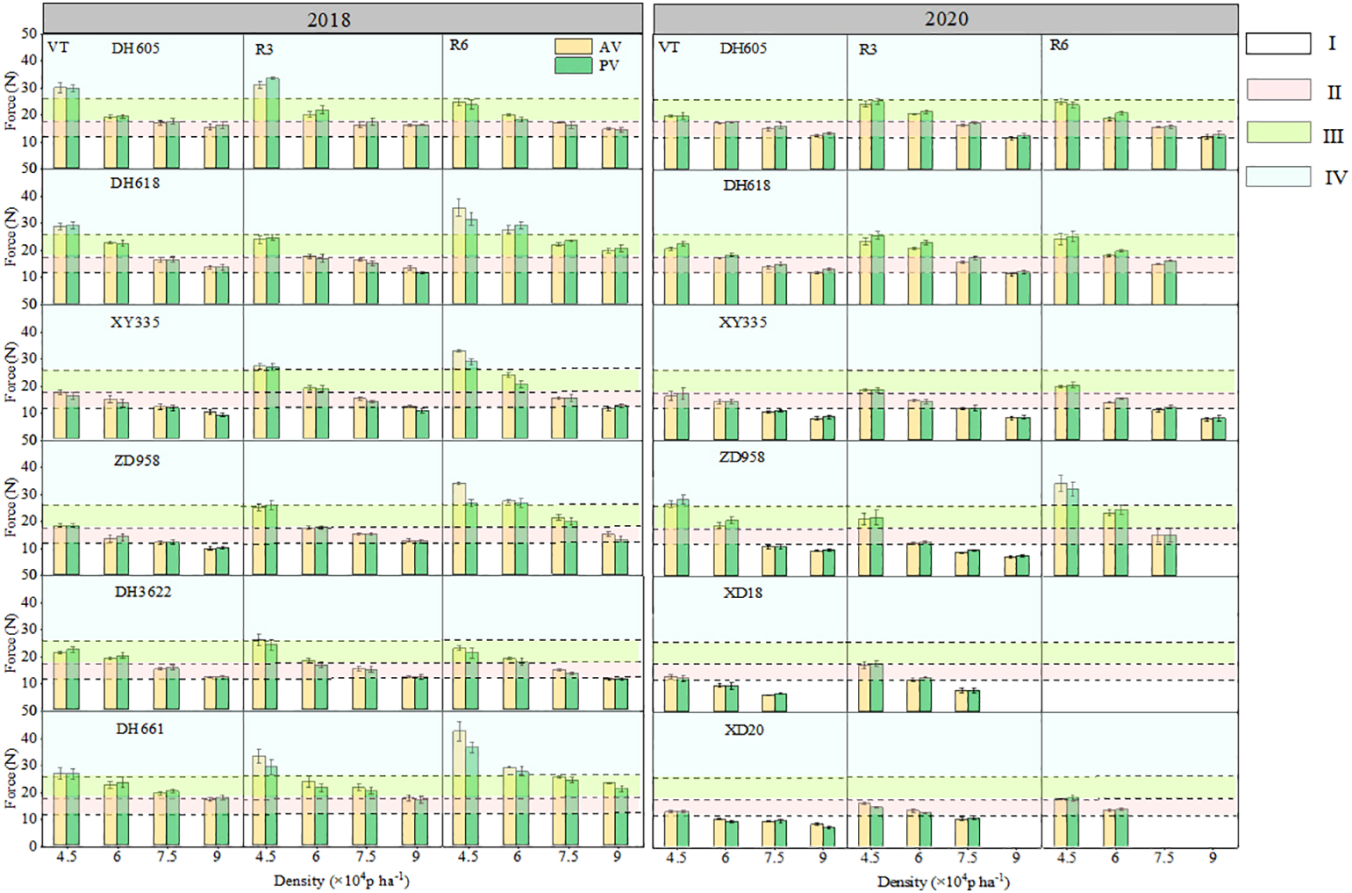
Measurement of lodging resistance of six varieties in different densities of maize plants. I: inferior lodging resistance, II: medium lodging resistance, III: high lodging resistance range, IV: strong lodging resistance. VT, flowering period; R3, grain filling period; R6, maturation period; AV, actual measured value *F*_max_ value; PV, presumptive value. The missing value of the force indicates that the angle at which the plant breaks is less than 45°.

The traits of plants from different varieties, planting density, and development period were principal factors for the lodging resistance of maize plants. The planting density usually is negatively associated with *F*_max_, that is, higher planting density usually led to a lower *F*_max_. Under low-density conditions, the differences of varieties are significant, that is, the data of lodging resistance from a population consisting of different varieties show great heterogeneity, while at high density, the differences between varieties become smaller. Maize plants at different developmental stages or in different reproductive periods show obvious differences in lodging resistance. From **Figure 6**, it is obvious that the *F*_max_ estimation and *F*_max_ measurement produced consistent results.

**Figure 7:**
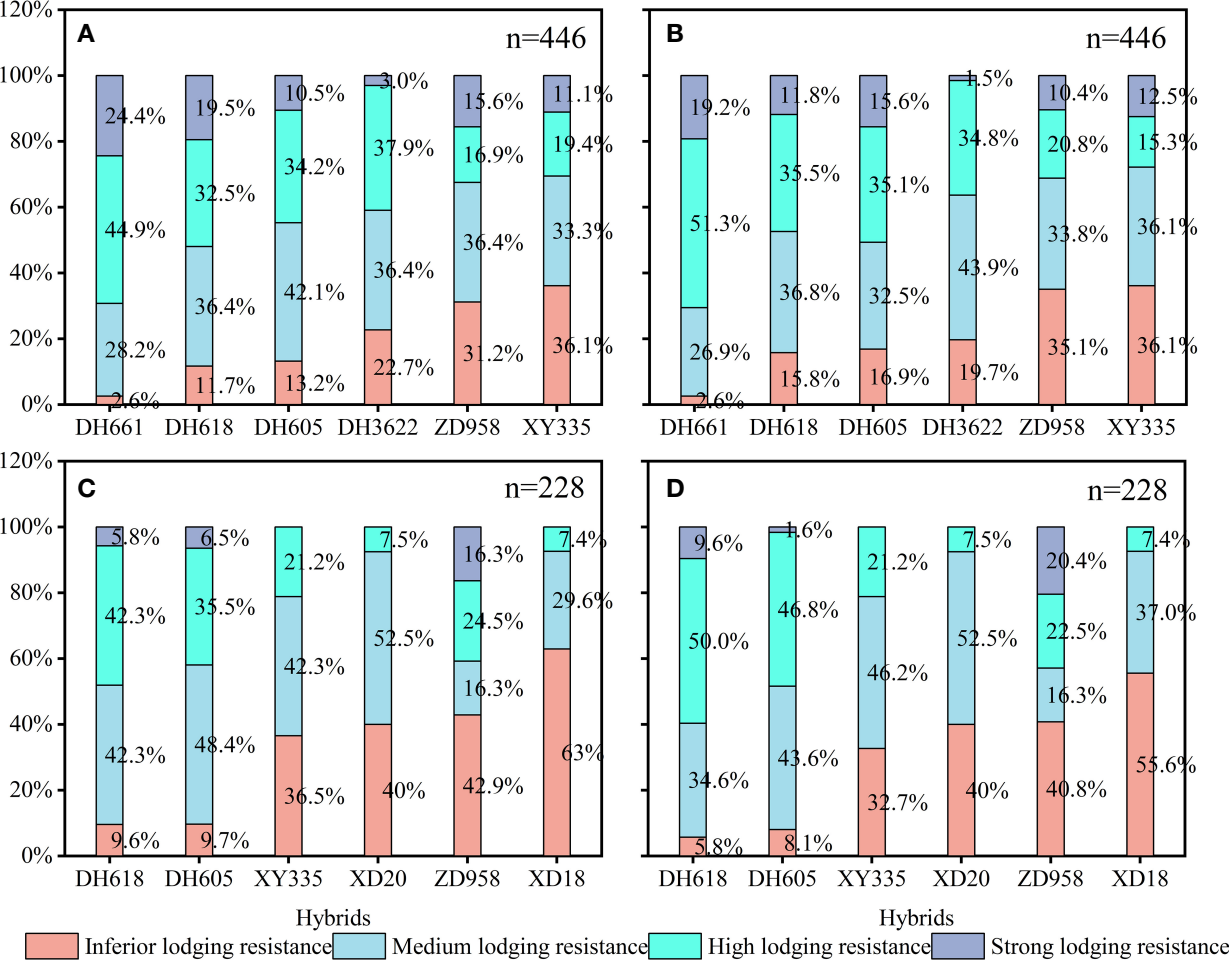
**(A)** In 2018, the actual measured value (AV) of all plants was rated for lodging resistance and the proportion of different grades of plants was determined. **(B)** In 2018, all plants were rated for lodging resistance based on presumptive value (PV) and the proportion of different grades of plants was determined. **(C)** In 2020, the AV of all plants was rated for lodging resistance and the proportion of different grades of plants was determined. **(D)** In 2018, all plants were rated for lodging resistance based on EV and the proportion of different grades of plants was determined.

The eight varieties of maize were rated according to their lodging resistance in different planting densities in 2018 and 2020. Based on *F*_max_, DH661, DH618, and DH605 were high lodging-resistant varieties, with inferior lodging resistance detected in only 2.6%, 10.7% and 11.5% of total plants in two years. DH3622, ZD958, XY335, XD20, and XD18 had poor lodging resistance, with 22.7%, 37.1%, 36.1%, 40.0%, and 63.0% of plants having inferior lodging resistance (**Figures 7A, C**). The same conclusion was obtained by using the F45 measurement method (**Figures 7B, D**).

## 4 Discussion

### 4.1 Advantages of non-destructive testing in the field

In 2019, Cook published a paper in which a lodging resistance tester, Darling, was developed. Darling was the first instrument for the lodging resistance measurement of crops in the field. The Darling tester produces a thrust at a fixed height to assess the lodging resistance of maize and sorghum, but actually evaluates the strength and toughness of the plant stalk. The Darling uses a destructive test for maximum lodging resistance.

The purpose of our test is to assess the lodging resistance of plants in a non-destructive way, which is important for the selection of commercial cultivars and cultivation of crops. For the lack of appropriate tools and test methods, the maximum lodging resistance of plants was determined and used to evaluate the maize lodging resistance, which is based on the stalk-breaking of plants and tensile strength from different angles, and inevitably causes the breaking of plants, resulting in maize plants that could not grow normally after the test. In order to evaluate maize lodging resistance in maize without injuring the plant, the maize stalk breaking rate and tensile value at different angles were determined. *F*_45_ gave an index of maize lodging resistance with a very low breaking rate, and from the correspondence between *F*_max_ and *F*_45_ (**Figure 5E**), the lodging resistance of plant was determined with *F*_45_. It is concluded that *F*_45_ can represent maize lodging resistance, which can be obtained by an NDT method in the fields. In this study, the *F*_45_ values were determined by a lodging resistance tester in the field and the lodging resistance of plants was calculated to predict the maximum lodging resistance, which achieved the *in situ* non-destructive testing of lodging resistance in maize.

### 4.2 Accuracy of non-destructive evaluation of maize lodging resistance in the field

This field non-destructive testing method can evaluate lodging resistance under different conditions. In 2018 and 2020, six varieties were selected to experiment on different densities, and the results show (1) among the varieties there existed significant differences in lodging resistance (DH661 > DH618 > DH605 > DH3622 > ZD958 > XY 335 > XD 20 > XD18); (2) the lodging resistances of plants from one variety were determined by differences in planting density, and higher planting density led to lower lodging resistance in all varieties; and (3) when the plants of one variety were tested for lodging resistance in different developmental periods, conclusions were usually consistent.

### 4.3 Reliability of the non-destructive *in situ* lodging resistance tester method in the field

At present the determination of maize lodging resistance usually uses the three-point bending method or the moving wind tunnel method. There are multiple instruments for the three-point bending method evaluation; the most popular tester is the YYD-1 lodging resistance tester produced by Zhejiang Topu Yunnong Technology Co., Ltd. With this instrument, DH661 (Ren et al., 2016) and DH618 showed strong lodging resistance (Ren et al., 2016; Gu et al., 2017; Yang et al., 2022), and XY335, ZD958, and XD20 showed weaker lodging resistance (Gu et al., 2017; Xue et al., 2020; Yang et al., 2022). By using digital detector FGJ-5, the stem breaking resistance of different varieties of maize was rated as XY335 > ZD958 > XD20, and the bending resistance of plant was ZD958 > XD20 (Cheng, 2010). By using a mobile wind tunnel to detect lodging resistance, Wen rated the wind resistance performance of different varieties as XY335 > ZD958 > XD20 (Wen et al., 2019). The results from non-destructive testing used in this study were nearly identical.

## 5 Conclusion

This non-destructive *in situ* method can test the lodging resistance of a maize stem by determining the angle values of bending plant in constant pull force, or reading the values of pull force on a plant that is inclined 45°°, based on the force strength on the plants in the field, composed of gravity and wind force. Non-destructive determination for maize lodging resistance in the field by using the lodging resistance tester can be successfully performed. The accuracy of the method was examined with different plants of 113 varieties for 3 years under different planting densities and developmental periods. *F*_45_ on maize plants at a 45° inclination was suitable to characterize maize lodging resistance in the field, and was the best index for the evaluation of maize lodging resistance in this study. According to the *F*_45_ value, the maximum lodging resistance *F*_max_ can be inferred, and the formula is *F*_max_ = 1.1354*F*_45_ – 0.3358.

## Data availability statement

The original contributions presented in the study are included in the article/supplementary material. Further inquiries can be directed to the corresponding authors.

## Author contributions

All authors have contributed significantly to the research. JSY: investigation, writing – original; XM, ZL, QY, and PZ: data curation, data analysis, visualization; SY: the inventor of the maize inversion resistance tester; JZY: the experimentalist in Weifang, sampling; XS: supervision; YW and LW: conceptualization, funding acquisition. All authors contributed to the article and approved the submitted version.
